# Acceleration of ultrafast demagnetization in van der Waals ferromagnet Fe_3_GeTe_2_ in high magnetic field

**DOI:** 10.1093/nsr/nwaf185

**Published:** 2025-05-23

**Authors:** Zhou Wang, Tao Sun, Zhongzhu Jiang, Mengju Yuan, Yuan Huang, Yifan Ren, De Hou, Tao Li, Xinyu Liu, Xuan Luo, Yisheng Chai, Alexey Kimel, Yuping Sun, Zhigao Sheng

**Affiliations:** High Magnetic Field Laboratory, Hefei Institutes of Physical Science, Chinese Academy of Sciences, Hefei 230031, China; University of Science and Technology of China, Hefei 230026, China; High Magnetic Field Laboratory, Hefei Institutes of Physical Science, Chinese Academy of Sciences, Hefei 230031, China; University of Science and Technology of China, Hefei 230026, China; Key Laboratory of Materials Physics, Institute of Solid State Physics, Chinese Academy of Sciences, Hefei 230031, China; Low Temperature Physics Laboratory, College of Physics, Chongqing University, Chongqing 401331, China; Advanced Research Institute of Multidisciplinary Science, Beijing Institute of Technology, Beijing 100081, China; High Magnetic Field Laboratory, Hefei Institutes of Physical Science, Chinese Academy of Sciences, Hefei 230031, China; University of Science and Technology of China, Hefei 230026, China; High Magnetic Field Laboratory, Hefei Institutes of Physical Science, Chinese Academy of Sciences, Hefei 230031, China; University of Science and Technology of China, Hefei 230026, China; High Magnetic Field Laboratory, Hefei Institutes of Physical Science, Chinese Academy of Sciences, Hefei 230031, China; High Magnetic Field Laboratory, Hefei Institutes of Physical Science, Chinese Academy of Sciences, Hefei 230031, China; Key Laboratory of Materials Physics, Institute of Solid State Physics, Chinese Academy of Sciences, Hefei 230031, China; Low Temperature Physics Laboratory, College of Physics, Chongqing University, Chongqing 401331, China; Institute for Molecules and Materials, Radboud University, Nijmegen 6525 AJ, The Netherlands; High Magnetic Field Laboratory, Hefei Institutes of Physical Science, Chinese Academy of Sciences, Hefei 230031, China; Key Laboratory of Materials Physics, Institute of Solid State Physics, Chinese Academy of Sciences, Hefei 230031, China; Collaborative Innovation Center of Advanced Microstructures Nanjing University, Nanjing 210093, China; High Magnetic Field Laboratory, Hefei Institutes of Physical Science, Chinese Academy of Sciences, Hefei 230031, China; Collaborative Innovation Center of Advanced Microstructures Nanjing University, Nanjing 210093, China

**Keywords:** 2D ferromagnets, Fe_3_GeTe_2_, demagnetization, magnetic field effect, spin entropy

## Abstract

The mechanisms of ultrafast laser-induced demagnetization of ferromagnets have been among the most heavily debated topics in ultrafast magnetism from the very beginning of the field. Here, we demonstrate that the timescale and the efficiency of ultrafast demagnetization of two-dimensional van der Waals ferromagnet Fe_3_GeTe_2_, excited by femtosecond laser pulses, can be efficiently accelerated by an external magnetic field. With a 1 T magnetic field at Curie temperature (*T*_C_) = 210 K femtosecond laser excitation causes demagnetization of the ferromagnet by 79% within 22.2 ps, while the application of the field at 7 T can suppress the demagnetization efficiency down to 52% and accelerate the process so that it is completed within 9.9 ps. We also reveal that the efficiency and the timescale can be varied in a similar way by changing the temperature of the sample, and the magneto-effect is more pronounced in the middle temperature region (90 to 210 K). Based on these observations we propose a thermodynamic explanation of the findings within the frames of a three-temperature model and without the involvement of any peculiarities to the electronic structure of van der Waals materials. Hence, our work emphasizes that controlling ultrafast demagnetization with the help of an applied magnetic field must be a general phenomenon, which is not limited to van der Waals materials, and thus must also be observed in other magnets.

## Teaser

Magnetic field can speed up ultrafast laser-driven demagnetization in a novel 2D magnet Fe3GeTe2, revealing a universal control mechanism for future high frequency tech.

## INTRODUCTION

The field of ultrafast magnetism started with the seminal discovery of ultrafast demagnetization of ferromagnetic (FM) Ni by femtosecond laser pulses [1]. The discovery revealed that FM order can be destroyed on a timescale much faster than the characteristic times of any interaction of spins with other reservoirs of angular momentum, such as free electrons and the lattice, known at that time. Naturally, the discovery caused an intense debate about the mechanisms of ultrafast demagnetization, where the thermodynamic three-temperature model turned out to be the most intuitive and, consequently, the most frequently employed one. The model as such does not take into account any peculiarities of the electronic or magnetic structure. Instead, it describes a magnet as three interconnected reservoirs of energy and angular momentum—electron, lattice and spin. Femtosecond laser excitation increases the temperatures of both electrons and the lattice thus initiating an exchange of heat between these two reservoirs and spins. As a matter of fact, with the exception of a very few experimental observations and theoretical predictions, most studies during the nearly 30-year-long history of ultrafast magnetism were performed in magnetic fields well below 1 T thus ignoring this degree of freedom in experimental research.

In 2017, stable FM ordering within monolayer limits had been discovered independently for the first time in the two-dimensional (2D) van der Waals (vdW) Cr_2_Ge_2_Te_6_ and CrI_3_ by Xiang Zhang and Xiaodong Xu *et al.* [2,3]. The natural vdW gap existing in these novel 2D magnets results in their high anisotropic properties, immediately enabling novel spin dynamic phenomena. For instance, Shan *et al.* achieved sub-THz spin wave excitation in a 2D A-type antiferromagnet CrI_3_ [4]. In our previous work, the longest remagnetization process to date (>3000 ps) has been observed in the 2D Cr_2_Ge_2_Te_6_ [5]. Recently, Lichtenberg *et al.* reported that when applying an external magnetic field below 0.35 T it was possible to control the characteristic time of ultrafast demagnetization of 2D ferromagnet Fe_3_GeTe_2_ (FGT) [6]. It was proposed that the dependence of the demagnetization time on the applied magnetic field was due to anisotropy in the spin relaxation time. Here, we focus on this effect and explore ultrafast laser-induced demagnetization in magnetic fields up to 7 T in the 2D vdW ferromagnet FGT. We found that both the timescale and the efficiency of ultrafast demagnetization in FGT can be significantly accelerated by an external magnetic field. With the increase of magnetic field from 1 to 7 T, the demagnetization time is accelerated from 22.2 to 9.9 ps and the demagnetization ratio caused by same laser power drops from 79% to 52%. A thermodynamic explanation of the findings within the framework of the three-temperature model is proposed, without involving any specific electronic structure peculiarities of van der Waals materials.

## RESULTS

### Structure and static magnetization of FGT

**Figure 1.**
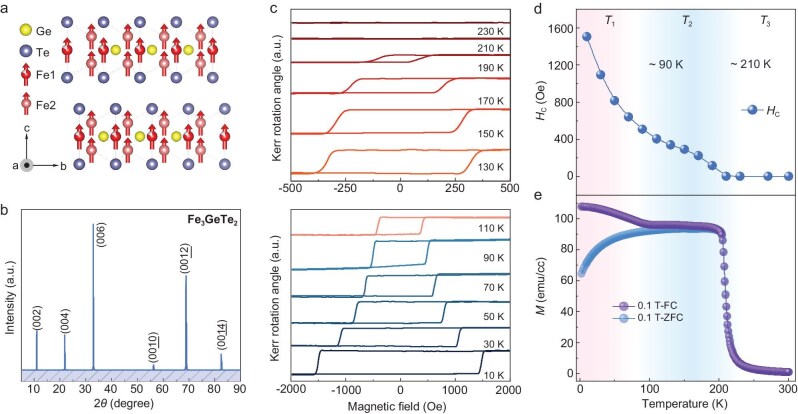
Structure and static magnetization of FGT. (a) Layered crystal structure diagram of an FGT crystal. (b) XRD analysis of FGT single crystal. (c) Kerr rotation angle *θ*_K_ as functions of magnetic field (along *c*-axis) measured at different temperatures. (d) Temperature-dependence of *H*_C_ (extracted from (c)) for FGT. (e) Temperature-dependence of magnetization for FGT measured with zero field cooling (ZFC) and field cooling (FC) modes (*H* = 0.1 T along *c*-axis).

Among 2D vdW magnets, Fe_3-x_GeTe_2_ stands out with a relatively high Curie temperature (*T*_C_) (ranging from 150 to 220 K depending on Fe occupancy) [7,8]. In this work, FGT was chosen for the spin dynamic study. Figure 1a shows the layered hexagonal structure of an FGT crystal with the space group P63/mmc (No. 194). The lattice parameters of the unit cell are *a* = *b* ≈ 4 Å and *c* ≈ 16 Å. Three Fe atoms occupy two inequivalent Wyckoff sites, designated as Fe1 and Fe2. Monolayer FGT has five sublayers (Te − Fe1 − (Fe2 & Ge) − Fe1 − Te) stacked along the *c*-direction, with Te atoms at the top/bottom, Fe1 in the second/fourth layers, and Fe2 & Ge at the center. The adjacent fivefold-layers are bonded by vdW forces with distance ∼2.95 Å, and can be cleaved along the (001) surface [9]. Figure 1b displays the X-ray diffraction (XRD) measurement results of the FGT flake studied here. There are only the (0 0 2*n*) Bragg peaks (n = 1, 2, 3, 4, 5, 6, 7) and no other peaks can be found in the XRD pattern, indicating the high-quality of the FGT samples.

Before studying the ultrafast spin dynamics of FGT, the static magnetization properties of FGT were first investigated using the polar magneto-optical Kerr effect (MOKE) technique, where a normally irradiated polarized laser was used (see Methods). When a laser irradiates on the surface of a bulk crystal, thermal gradient issues can arise, complicating the laser-induced phenomena. To avoid this complexity, we employ a FGT thin film with a thickness of 5 nm in this work, which is fabricated through mechanical exfoliation using a gold auxiliary [10]. Figure 1c shows the Kerr rotation angle (*θ*_K_) as a function of the magnetic field measured at different temperatures. As the temperature increases, the hysteresis loop shrinks and the coercive field (*H*_C_) decreases. As shown in Fig. 1d, the *H*_C_ decreases quite fast from 10 to 90 K, and then decreases a little bit more slowly when *T* >90 K. Quantitatively, the *H*_C_ decreases by ∼68% from 1499 Oe at 10 K to 482 Oe at 90 K, and then turns to zero ∼210 K, which corresponds to the Curie temperature (*T*_C_) of FGT.

The static magnetization properties of FGT have also been explored by a superconducting quantum interference device (SQUID) magnetometer (details in Methods). Figure 1e shows the ZFC and FC magnetization curves obtained with the external magnetic field *H* = 0.1 T along the *c* axis. A paramagnetic to FM transition is observed at *T*_C_ ∼210 K, consistent with the MOKE results and previous reports [11]. Moreover, it is interesting to find that the magnetization curves of ZFC and FC diverge near 90 K. The ZFC curve drops down when *T* <90 K, indicating the presence of antiferromagnetic (AFM) spin coupling in 5 nm thick FGT film [9,11–15]. Further magnetic field dependent magnetization measurement (*M-H*) shows that such a canted AFM phase can be switched to FM at a critical magnetic field (∼200 Oe @ 11 K; Supplementary Information S1). Based on the static magnetization measurement results (both MOKE and SQUID), the magnetic state of FGT can be divided into three temperature zones (Fig. 1d). These are *T*_1_ (<90 K) in which the AFM and FM spin coupling coexist, *T*_2_ (90 to 210 K) in which FM spin coupling is dominant, *T*_3_ (>210 K) in which FGT is in the paramagnetic phase.

To explore the spin-lattice interaction of FGT, the static magnetostriction effect has been explored. It is found that within the saturation magnetic field (*H*_S_), the domain wall motion in the FGT crystal contributes to magnetostriction and a sharp change of the magnetostriction coefficient dλ′/d*H* is found near the *H*_C_ (S2a). Above *H*_S_, the magnetic field has a negligible effect on dλ′/d*H* at lower temperatures due to the freezing of thermal energy [16]. Near *T*_C_ an increase in the magnetic field would cause a decrease of dλ′/d*H* (S2b), which will be further discussed later.

### Temperature dependence of ultrafast demagnetization in FGT

**Figure 2.**
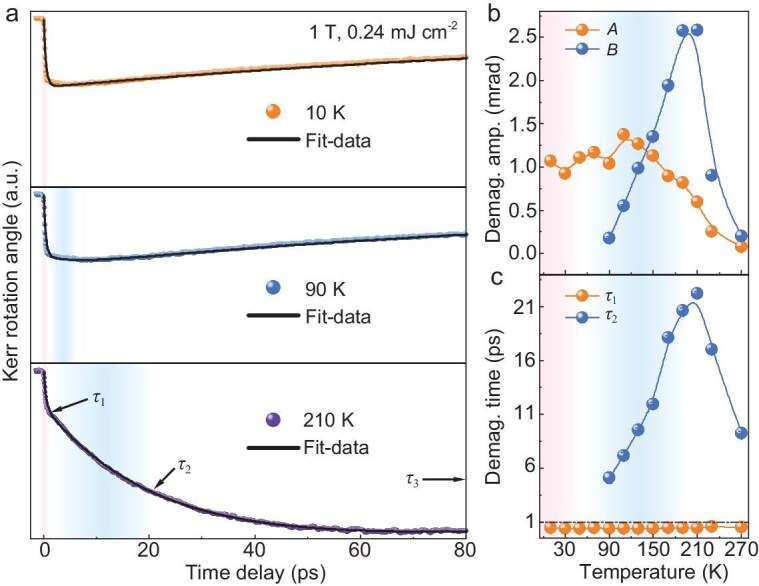
Temperature dependence of spin dynamics in FGT. (a) Time-resolved Kerr rotation angle as a function of pump-probe delay time in FGT measured at different temperatures with a magnetic field of 1 T (along *c*-axis). (b) Temperature-dependent demagnetization amplitudes of the Type I (*A*) and Type Ⅱ (*B*) demagnetization processes. (c) Temperature-dependent demagnetization times of the Type I (*τ*_1_) and Type Ⅱ (*τ*_2_) demagnetization processes.

After exploring the static magnetization, we now turn to the ultrafast spin dynamics in the 2D vdW FGT crystal. Time-resolved polar MOKE (TR-MOKE) spectroscopy was employed to probe the ultrafast spin dynamics of FGT thin film. Initially, the ambient temperature dependence of the spin dynamic was measured with a fixed magnetic field of 1 T along the [001] direction. It is observed that the ultrafast demagnetization process of FGT exhibits a strong temperature dependence (S3). As shown in Fig. 2a, after laser pumping the *θ*_K_ measured at 10 K drops 0.78 mrad quickly within 0.5 ps and then gradually recovers, which is a typical fast demagnetization process (Type I) [17,18]. Such a fast demagnetization phenomenon is maintained in the low temperature region (*T*_1_ region). When the sample is warmed up to the *T*_2_ region (*T* >90 K), an additional slow demagnetization process emerges. The *θ*_K_ drops 0.66 mrad fast within 0.4 ps at first and then continuously drops down to 1.11 mrad within 5.1 ps. Such a two-step process is the typical feature of Type Ⅱ demagnetization [17,19,20]. This Type II demagnetization dynamic is more pronounced and slow demagnetization takes longer when the temperature is close to the *T*_C_.

The key characteristics of ultrafast demagnetization are demagnetization time and amplitude. To quantitatively examine the two-step demagnetization times and magnetization losses of FGT, the experimental results are fitted using the equation below [17]:


(1) \begin{eqnarray*}
{\theta }_K\left( t \right) = \left( {A \cdot \left( {1 - {e}^{\frac{{ - t}}{{{\tau }_1}}}} \right) + B \cdot \left( {1 - {e}^{\frac{{ - t}}{{{\tau }_2}}}} \right)} \right) \cdot {e}^{\frac{{ - t}}{{{\tau }_3}}},
\end{eqnarray*}

where *A* and *B* represent the amplitudes of the first- and second-step demagnetizations, respectively; *τ*_1_ and *τ*_2_ correspond to the fast and slow demagnetization times, respectively; *τ*_3_ are the timescales of remagnetization. According to the mathematical formulation of Equation (1), the demagnetization process can be interpreted as the superposition of two exponential curves on different timescales. By fitting the experimental data, the specific demagnetization amplitudes and timescales at different temperatures were obtained and are summarized in Fig. 2b and c. It can be observed that both the Type I demagnetization amplitude *A* and the Type II demagnetization amplitude *B* have different temperature behavior. In the *T*_1_ region (*T* <90 K), *A* increases gradually with of the increase in temperature, while *B* is zero. Within the *T*_2_ region (*T* >90 K), *A* decreases slowly, while B begins to increase rapidly reaching 2.58 ± 0.01 mrad around *T*_C_ ∼210 K. When *T* >210 K, both *A* and *B* drop and eventually drop to zero near ∼300 K. In addition to the demagnetization amplitudes (*A* and *B*), the demagnetization times (*τ*_1_ and *τ*_2_) also behave differently upon a temperature increase. As shown in Fig. 2c, the fast demagnetization time (Type I) *τ*_1_ is nearly temperature independent. While the slow demagnetization time (Type II) *τ*_2_ increases from 5.12 ± 0.50 ps at *T* ∼90 K to 22.20 ± 0.18 ps at *T* ∼210 K. Moreover, it is interesting to find that the remagnetization time *τ*_3_ has similar temperature dependence as those of *B* and *τ*_2_ (S4).

### Magnetic field effect on the demagnetization of FGT

**Figure 3.**
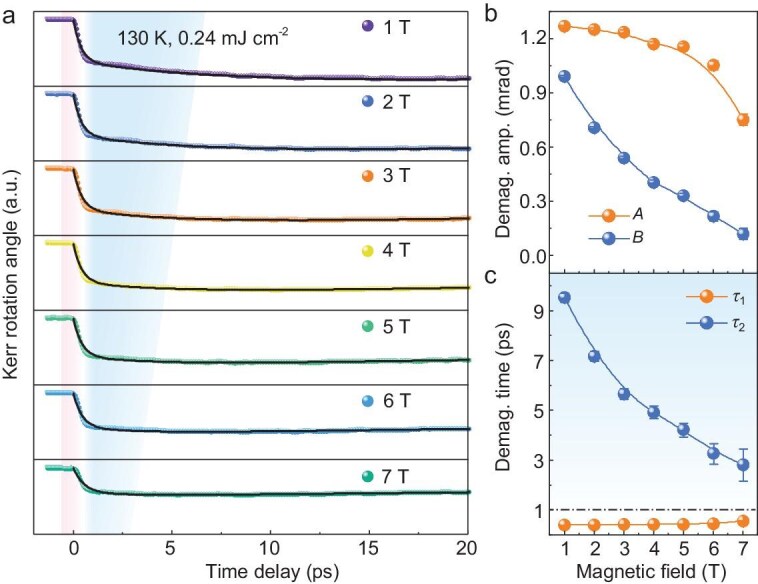
Magnetic field effect on the demagnetization of FGT. (a) Time-resolved Kerr rotation angle as a function of pump-probe delay time in FGT flake measured at 130 K with different magnetic fields (along *c*-axis). (b) Magnetic-field-dependent demagnetization amplitudes of the Type I (*A*) and Type Ⅱ (*B*) processes. (c) Magnetic-field-dependent demagnetization times of the Type I (*τ*_1_) and Type Ⅱ (*τ*_2_) processes.

As a 2D vdW magnetic material, the magnetism of FGT is sensitive to the external magnetic field [2,9]. Next, the spin dynamic behavior of FGT was explored under different magnetic fields. According to the temperature dependence behavior, the magnetic field effects in different temperature regions have been carefully examined. In the *T*_1_ region (*T* <90 K), the external magnetic field can slightly reduce the demagnetization amplitude *A*, the timescale *τ*_1_ as well as the remagnetization time *τ*_3_ (S5). For instance, a 7 T magnetic field causes the demagnetization amplitude (*A*) to decrease from 1.105 ± 0.002 to 0.915 ± 0.003 mrad with a reduction ratio of −17% and the demagnetization timescale (*τ*_1_) decreases from 0.415 ± 0.007 to 0.322 ± 0.011 ps with a reduction ratio of −23%, respectively. In the *T*_2_ region (*T* >90 K), this magnetic field effect is more significant. The typical TR-MOKE data obtained at 130 K are shown in Fig. 3a. It can be found that, with an increase of the magnetic field from 1 to 7 T, the magnetic field can reduce the demagnetization amplitude (*A + B*) from 2.25 ± 0.02 to 0.87 ± 0.06 mrad with a reduction ratio of −61%. At the same time, the magnetic field can reduce the whole demagnetization timescale (*τ*_s_ = *τ*_1_  *+ τ*_2_) from 9.52 ± 0.19 to 2.82 ± 0.63 ps with a reduction ratio of −70%. This is the most striking feature of this work, which clearly indicates that the magnetic field can accelerate the demagnetization process in 2D vdW magnet FGT.

It is worth noting at this point that the magneto-acceleration effects observed in the *T*_2_ region also differ for different demagnetization processes. In detail, the first-step demagnetization process has a small response, while second-step demagnetization is highly sensitive to the magnetic field. The demagnetization amplitudes and timescales obtained at different magnetic fields are summarized in Fig. 3b and c, respectively. For the first-step demagnetization process, with an increase in the magnetic field from 1 to 7 T, the demagnetization amplitude *A* decreases from 1.26 ± 0.01 to 0.75 ± 0.03 mrad with a relative change ratio -40%, and the *τ*_1_ increases from 0.39 ± 0.01 to 0.55 ± 0.03 ps with relative change ratio +41%. As for the second-step demagnetization process, the amplitude *B* decreases from 0.99 ± 0.01 to 0.12 ± 0.03 mrad and the *τ*_2_ drops from 9.52 ± 0.19 to 2.82 ± 0.63 ps when the magnetic field increases from 1 to 7 T. The relative change is as high as −88% and −70% for *B* and *τ*_2_, respectively. These results clearly reveal that the magneto-acceleration effect is more significant in the slower demagnetization process. To explore the universality of magneto-acceleration phenomena, additional TR-MOKE experiments with magnetic field deflected 45 degrees from the *c*-axis of the FGT sample have been conducted, and the typical results are summarized in S6. It is found that the magneto-acceleration effect can also be observed, and the relationship between the acceleration effect and the magnetic field under different experimental geometries are almost the same as each other (S6).

**Figure 4.**
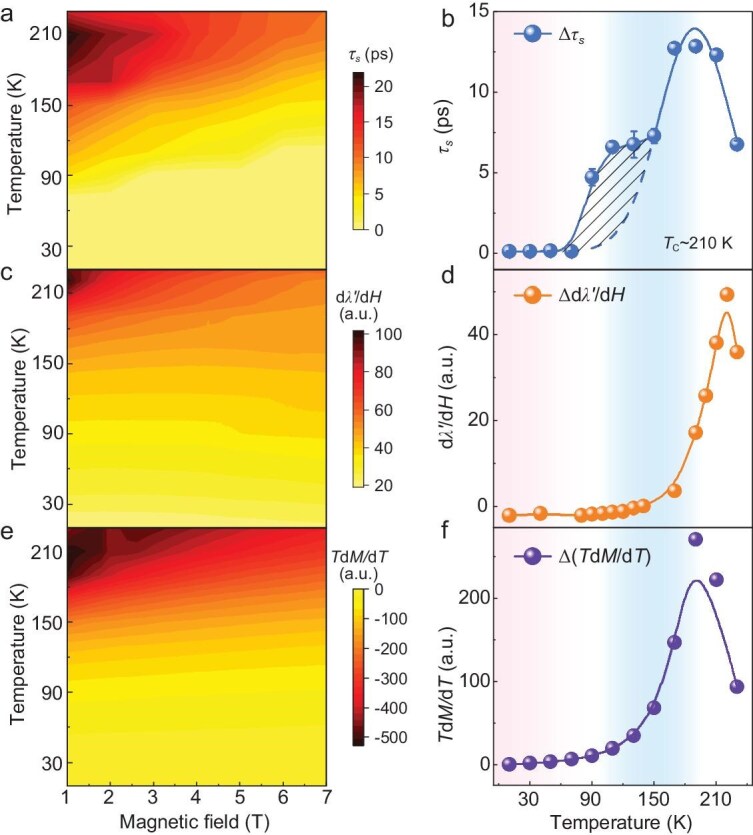
Phase diagrams of temperature-magnetic field effects. (a, c, e) Phase diagrams of temperature-magnetic field effects on *τ*_s_, dλ′/d*H* and *T*d*M*/d*T*, respectively. (b, d, f) Temperature dependence of ∆*τ*_s_, ∆dλ′/d*H* and ∆(*T*d*M*/d*T*), respectively. The magnetic-field-induced change ∆*R* (*R*=*τ*_s_, dλ′/d*H* and *T*d*M*/d*T*) is calculated as ∆*R* = *R*|_7T_ − *R*|_1T_. Twill shadow is the area of demagnetization anomalies.

According to the results shown above, both temperature and magnetic field can influence the demagnetization process in 2D vdW magnet FGT. Figure 4a summarizes both temperature and magnetic field effects on demagnetization timescales as a 2D phase diagram. At a fixed magnetic field, the demagnetization time *τ*_s_ increases with an increase of temperature and reaches its maximum near *T*_C_. With a fixed temperature, the demagnetization process can be accelerated step by step with an increase of the external magnetic field. The magnetic field-induced change of demagnetization time Δ*τ*_s_ (=*τ*_s_|_7T_ − *τ*_s_|_1T_) is calculated and its temperature dependence is shown in Fig. 4b. The Δ*τ*_s_ is small (<0.1 ps) at the *T*_1_ region (*T* <90 K) and increases rapidly when *T* >90 K. Such a magnetic field effect reaches its maximum around *T*_C_. At 210 K, with a magnetic field increase from 1 to 7 T, the demagnetization amplitude can be suppressed from 3.17 ± 0.01 down to 2.08 ± 0.02 mrad, and the demagnetization timescale can be accelerated from 22.20 ± 0.18 to 9.95 ± 0.13 ps with Δ*τ*_s_ ∼12.25 ps. The detailed magneto-effect on both demagnetization amplitudes and timescales can be found in S7.

### The origin of magnetic field effect on laser-induced demagnetization

The intriguing phenomenon observed above is that the high magnetic field can efficiently manipulate both the amplitude and timescale of demagnetization and such a magneto-effect is more pronounced for the Type II process. It is believed that the slow demagnetization is closely related to the spin-lattice interaction and the phonons transfer angular momentum to spins within this slow process [17]. In this context, the magnetic field effect on the lattice as well as the spin-lattice interaction should be considered in the observed magneto-acceleration effect. As mentioned above, magnetostriction has been studied carefully. Figure 4c summarizes all obtained magnetostriction coefficient dλ′/d*H* data as a temperature-magnetic field diagram and the temperature dependent magnetostriction coefficient Δdλ′/d*H* (=dλ′/d*H*|_7T_ − dλ′/d*H*|_1T_) is presented in Fig. 4d. It should be noted that the magnetostriction measurements system itself has a background that dλ'/d*H* increases linearly with increasing temperature. When comparing Fig. 4a and c, it is found that the magnetic field dependent τ_s_ and dλ′/d*H* show the opposite trend below 150 K. Moreover, the temperature dependent behavior of the Δ*τ*_s_ and Δdλ′/d*H* are quite different from each other (Fig. 4b and d), which implies that the magnetostriction is not the origin of the observed magneto-acceleration effect and other dominant factors should be considered.

Based on M3TM, ultrafast demagnetization is a process driven by heat exchange between electron, spin, and lattice systems. In the model, femtosecond laser pulses increase the temperatures of both electrons and lattice with respect to the spin temperature. Such a difference must be equilibrated by exchanging heat between all these reservoirs. The slower demagnetization process [17], which we manage to control with the help of an applied magnetic field, corresponds to the heat exchange between spins and lattice. The corresponding time of demagnetization, is given by *τ*_s_ = *C*_s_/*g*_sl_, where *g*_sl_ represents the efficiency of the spin-lattice interaction and *C*_s_ is the heat capacity of spins [21,22]. As the heat capacity, in general, is defined as *C*_s_ = d*Q/*d*T*, in terms of entropy, it can be rewritten as *C*_s_ = *T*(d*S/*d*T*). Expressing the entropy of the spin system in terms of the Taylor series concerning the magnetization *M*, we realize that the entropy should be an even function of the magnetization *M*. Any odd terms are forbidden by symmetry because the entropy is invariant with respect to time-reversal whilst magnetization is not. In the lowest order, we assume that *S* ∼ *M*^2^, which gives *C*_s_ ∼2*T*(d*M/*d*T*) and *τ*_s_ ∼2*T*(d*M/*d*T*)/*g*_sl_. Accordingly, with fixed spin-lattice interaction *g*_sl_, higher temperature induces slower demagnetization time and *τ*_s_ would get its maximum near *T*_C_ due the largest value of d*M/*d*T*, which quantitatively agrees with our observation as shown in Figs 2c and 4e.

By applying an external magnetic field, one aligns the spins and thus efficiently reduces the susceptibility of the magnetization to temperature. To examine the magnetic field effect on the spin heat capacity *C*_s_ as well as the *τ*_s_, detailed magnetization measurements have been conducted in FGT crystals (see Methods). Figure 4e summarizes all obtained *T*(d*M/*d*T*) data as a temperature-magnetic field diagram. It can be found that the external magnetic field can efficiently reduce the value of *T*(d*M/*d*T*) and such magneto-reduction increases almost linearly with an increase of the magnetic field even if the magnetic field is larger than *H*_C_. With a fixed magnetic field, the magneto-reduction effect increases with temperature and becomes significant around the *T*_C_. Figure 4f displays the temperature dependence of field-induced change Δ*T*(d*M/*d*T*) (=*T*(d*M/*d*T*)|_7T_ − *T*(d*M/*d*T*)|_1T_). It is clear that the magnetic field effect on the *T*(d*M/*d*T*) is quite similar to those on the demagnetization time Δ*τ*_s_ (Fig. 4b). In temperature region *T*_1_, the magnetic field effect is negligible. While it becomes significant in the *T*_2_ region and Δ*T*(d*M/*d*T*) increases with the enhancement of temperature. It reaches its maximum near *T*_C_, and then drops down when the temperature enters the *T*_3_ region. These results indicate that applying an external magnetic field aligns the spins and decreases the sensitivity of the magnetization to a temperature increase (d*M/*d*T* decreases) as well as the spin heat capacity *C*_s_ and the demagnetization time *τ*_s_.

Compared with Fig. 4b and f, it is interesting to find that the temperature dependent Δ*τ*_s_ and Δ*T*(d*M*/d*T*) do not match perfectly from 90 to 150 K, which has been marked as a twill shadow region in Fig. 4b. Actually, such an abnormal feature can also be found in the temperature dependent *τ*_2_ as shown in Fig. 2e. According to the static magnetization analysis mentioned above, there is canted AFM spin coupling in the FGT at *T* <90 K. When *T* >90 K, the canted AFM coupling starts to transfer to FM type. The abnormal ultrafast demagnetization occurring between 90 K and 150 K may be related to such magnetic phase transition, which intrinsically affects the spin entropy as well as the spin heat capacity *C*_s_ of FGT.

The acceleration of ultrafast demagnetization processes has been observed in magnetic heterojunctions. For example, Kuiper *et al.* conducted a comparative study on Co thin films and Co/Pt multilayers, finding that the presence of Co/Pt interfaces results in a 3-fold increase in demagnetization speed due to spin-orbit interactions [23]. Similarly, Nikolić *et al.* reported that demagnetization in the Ni/Pt bilayer is faster than in the Ni layer alone. Additionally, they found that this accelerated demagnetization enhances the magnitude of laser pump-induced THz emission in the Ni/Pt bilayer [24]. These studies achieved accelerated demagnetization by creating interfaces with strong spin-orbit coupling through the addition of a Pt layer to ferromagnetic metals. In our work, accelerated demagnetization is achieved in a single 2D ferromagnetic film by suppressing spin entropy using a high magnetic field. This material-independent method of regulating spin dynamics through an external magnetic field may be extended to other ultrafast dynamic processes. Moreover, this magneto-controlling dynamic may produce many interesting and useful emergent phenomena, such as THz emission enhancement, which merit further investigation in future studies.

## CONCLUSION

In this study, we explored the effect of magnetic field on the process of ultrafast laser-induced demagnetization of 2D vdW ferromagnet FGT in fields up to 7 T and in a broad range of temperatures. The timescale and efficiency of ultrafast demagnetization can be effectively controlled by both external high magnetic fields and temperature. For instance, applying a magnetic field with a strength of 7 T can substantially accelerate demagnetization from 22.2 ps to 9.9 ps. It is interesting to find that despite the dramatic field-induced changes, the effect can still be explained in terms of a simple three-temperature model which ignores any peculiarities of electronic and magnetic structure of materials. This fact demonstrates that the effect of magnetic field on laser-induced demagnetization is quite general, not limited to 2D vdW materials and must be observed in other types of magnets.

## METHODS

### TR-MOKE measurements

The ultrafast demagnetization curves of FGT were measured by the TR-MOKE method using a pump-probe technique. Samples were cooled in an Oxford 7T SpectromagPT cryostat (1.5–300 K, 0–7 T). A Ti:sapphire laser (800 nm, 150 fs, 1 kHz) generated pump/probe beams split by a 3:7 plate. The probe beam was frequency-doubled to 400 nm via a β-BaB_2_O_4_ crystal. Pump (400 μm spot, 0.24 mJ· cm^2^) and probe (300 μm spot, 0.06 mJ· cm^2^) beams were incident at 10° and 5°, respectively, with spot sizes verified by a Newport LBP2-HR-VIS2 analyzer. A motorized delay stage was used to control pump-probe timing (Δ*t*). TR-MOKE measurements applied perpendicular magnetic fields, detecting Kerr rotation at 630 Hz via a balanced photodetector. Magnetic signals were isolated by subtracting antiparallel field responses [Signal(+) − Signal(−)] to cancel non-magnetic artifacts.

### Magnetostriction measurements

In the composite magnetoelectric method, FGT, as the magnetostrictive phases, were glued onto a 0.2-mm-thick piezoelectric layer of a 0.7Pb(Mg_1/3_Nb_2/3_)O_3_ − 0.3PbTiO_3_ (PMN-PT) [001]-cut single crystal. Ag epoxy (H20E, Epoxy Technology Inc.) acted as the electrode and a strain mediator. Before electrical measurements, the PMN-PT was electrically poled under an electric field of 550 kV/m. The electrical signal of ME laminates *V_ME_* = *V*_x_ + i*V*_y_ (*V*_x_ and *V*_y_ represent in-phase and out-of-phase signals, respectively) was measured by a lock-in amplifier (OE1022, SYSU Scientific Instruments) with a commercial sample stick (MultiField Tech.). An AC magnetic field *H*_ac_ = 1 Oe was generated by a Helmholtz coil.

### Magnetization measurements

The magnetic properties of the FGT crystal were characterized using a Quantum Design MPMS3 SQUID-VSM system. Spontaneous magnetization measurements involved: (1) heating to 300 K (above *T*_C_) for 5 min, (2) zero-field cooling to 2 K (2 K/min) while recording VSM moment data. *M-T* curves were acquired at 2 K/min with 1 K intervals; *M-H* loops used 200 Oe/s sweep rates and 500 Oe steps. Both measurements employed 200 averages per data point.

## Supplementary Material

nwaf185_Supplemental_File
