# Supplementary material for: Acceleration of ultrafast demagnetization in van der Waals ferromagnet Fe3GeTe2 in high magnetic field
Source: Natl Sci Rev. 2025 May 23;12(7):nwaf185. doi: 10.1093/nsr/nwaf185 (PMC12218190; doi:10.1093/nsr/nwaf185)
Supplement: nwaf185_Supplemental_File [file nwaf185_supplemental_file.docx]

Supplementary Materials for

**Acceleration of ultrafast demagnetization in van der Waals ferromagnet Fe_3_GeTe_2_ in high magnetic field**

Zhou Wang^1,2,†^, Tao Sun^1,2,†^, Zhongzhu Jiang^3^, Mengju Yuan^4^, Yuan Huang^5^, Yifan Ren^1,2^, De Hou^1,2^, Tao Li^1^, Xinyu Liu^1^, Xuan Luo^3^, Yisheng Chai^4^, Alexey Kimel^6,*^, Yuping Sun^1,3,7^, Zhigao Sheng^1,7,*^

*^1^ High Magnetic Field Laboratory, HFIPS, Anhui, Chinese Academy of Sciences, Hefei 230031, China*

*^2^ University of Science and Technology of China, Hefei 230026, China*

*^3^ Key Laboratory of Materials Physics, Institute of Solid State Physics, HFIPS, Chinese Academy of Sciences, Hefei 230031, China*

*^4^ Low Temperature Physics Laboratory, College of Physics, Chongqing University, Chongqing 401331, China*

*^5^ Advanced Research Institute of Multidisciplinary Science, Beijing Institute of Technology, Beijing 100081, China*

*^6^ Institute for Molecules and Materials, Radboud University, Heyendaalseweg 135, 6525 AJ, Nijmegen, The Netherlands*

*^7^**Collaborative Innovation Center of Advanced Microstructures Nanjing University, Nanjing 210093, China*

^†^ These authors contributed equally to this work.

*** Corresponding authors: Z.C. (zhigaosheng@hmfl.ac.cn), A.K. (aleksei.kimel@ru.nl)

**This file contains:**

**Figure S1-S8**

Characterization of magnetic properties of FGT with ZFC mode, magnetostriction measurements data of FGT, TR-MOKE data and fit data of FGT.


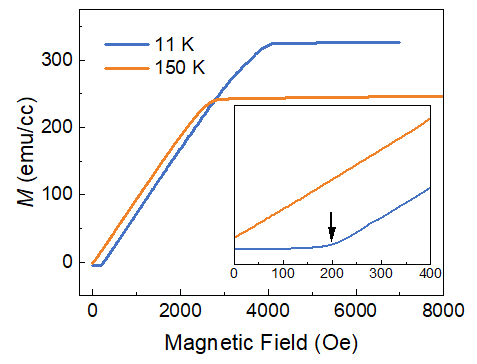


**Figure S1.** Magnetic field dependent of magnetization for FGT with zero-field-cooling (ZFC) modes measured *T*=11 K and 150 K along the *c*-axis.

**
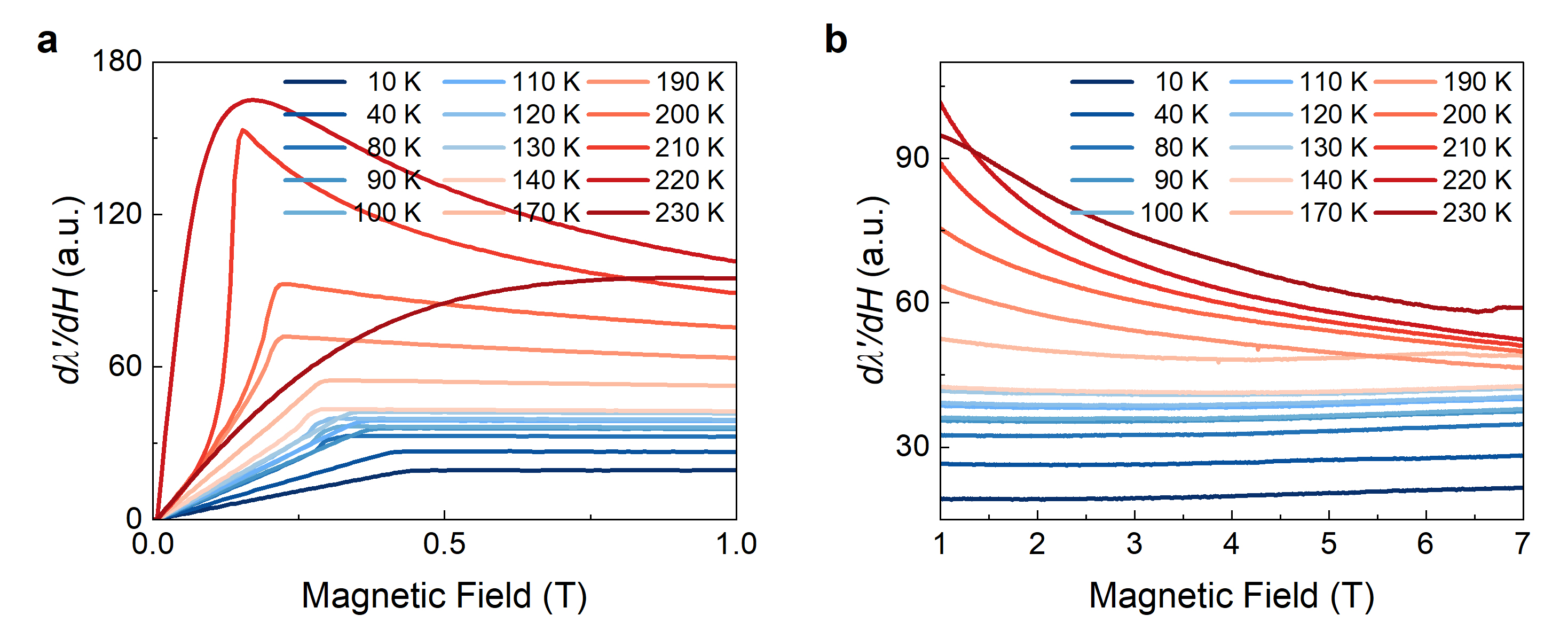
**

**Figure S2.** (a) The magnetic field (0 - 1 T) dependence of magnetostriction coefficient *dλ'*/*dH* measured by FGT at selected temperature. (b) The magnetic field (1 - 7 T) (along *c*-axis) dependence of magnetostriction coefficient *dλ'*/*dH* measured by FGT at selected temperature.


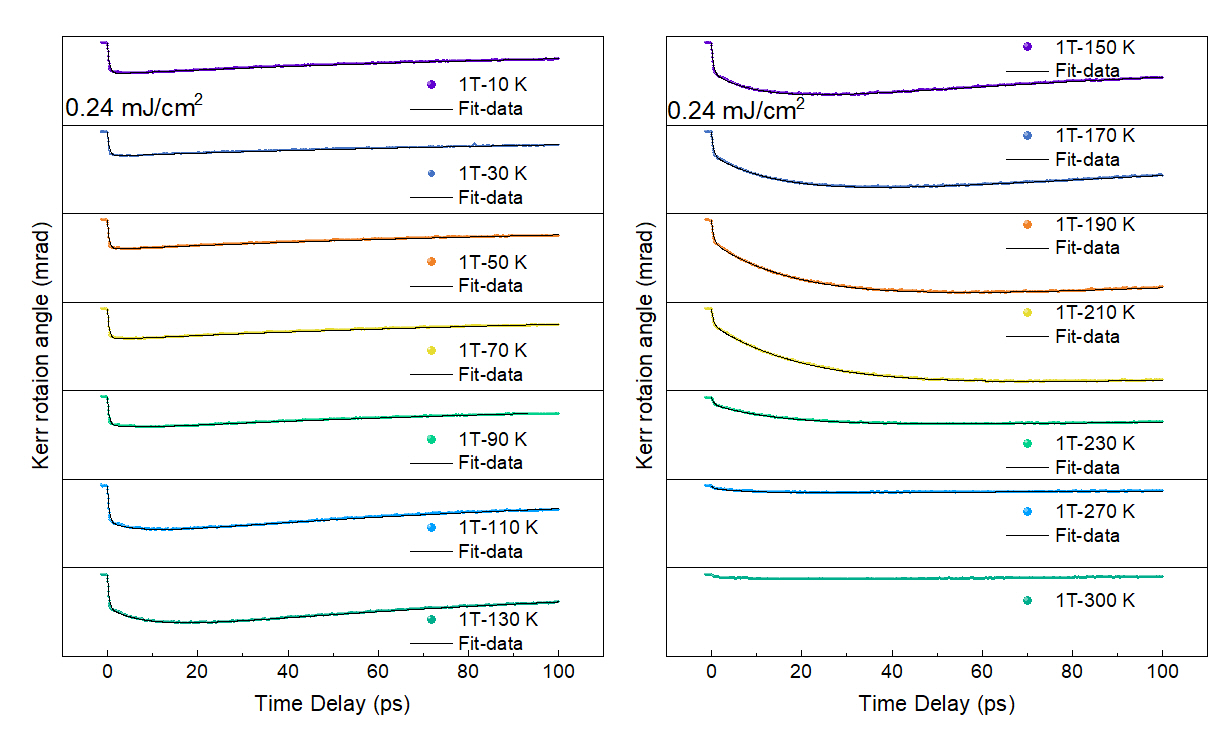


**Figure S3.** Time-resolved Kerr rotation angle as a function of pump–probe delay time in thin-layer FGT flake under different temperature with 1 T magnetic field (along *c*-axis).


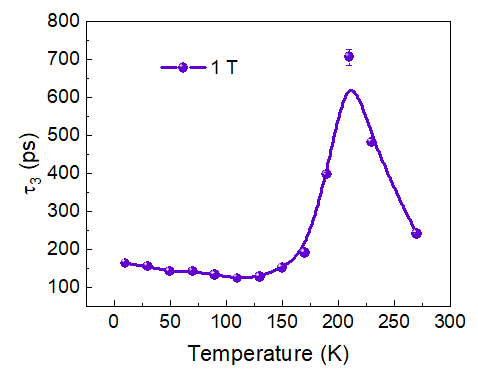


**Figure S4.** Temperature dependence of remagnetization time scale *τ*_3_ obtained from raw shown in **S3**.


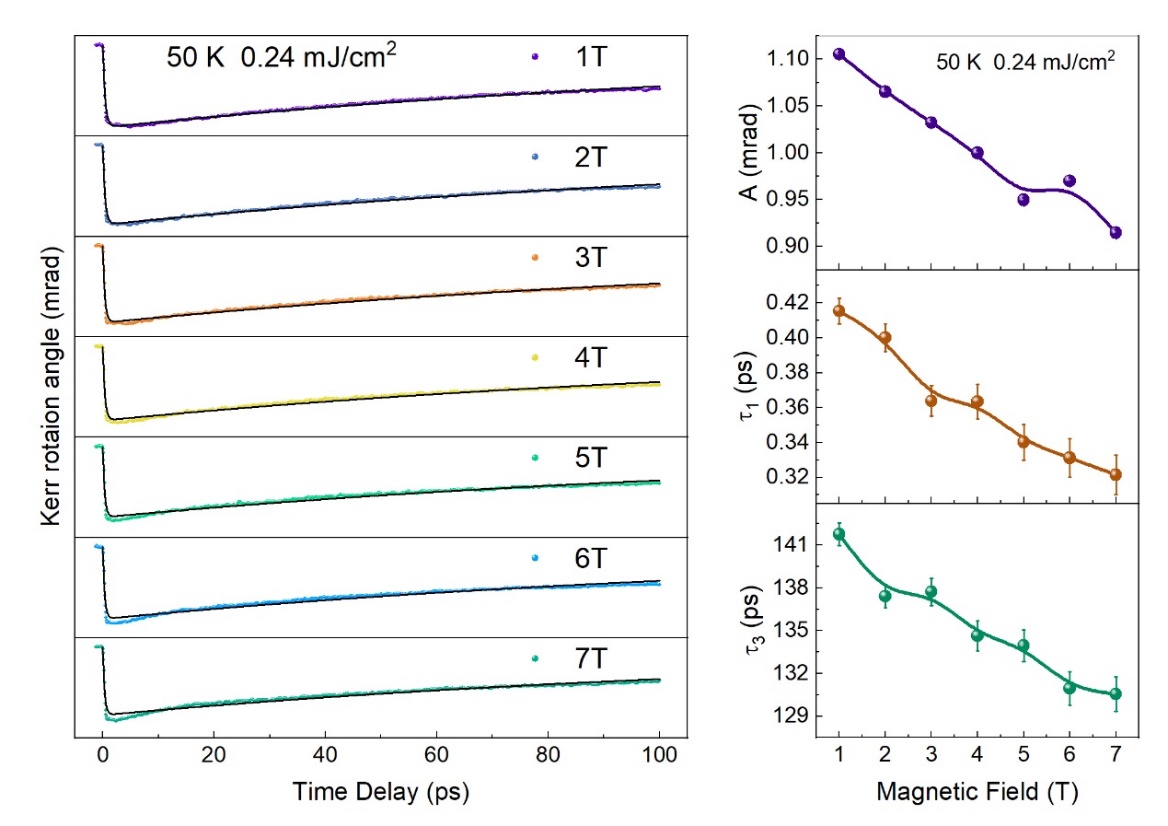


**Figure S5.** Time-resolved Kerr rotation angle as a function of pump–probe delay time in thin-layer FGT flake under different magnetic field (along *c*-axis) at 50 K. Magnetic field-dependence of *A*, *τ*_1_ and *τ*_3_ extracted from the fit shown in the original data.


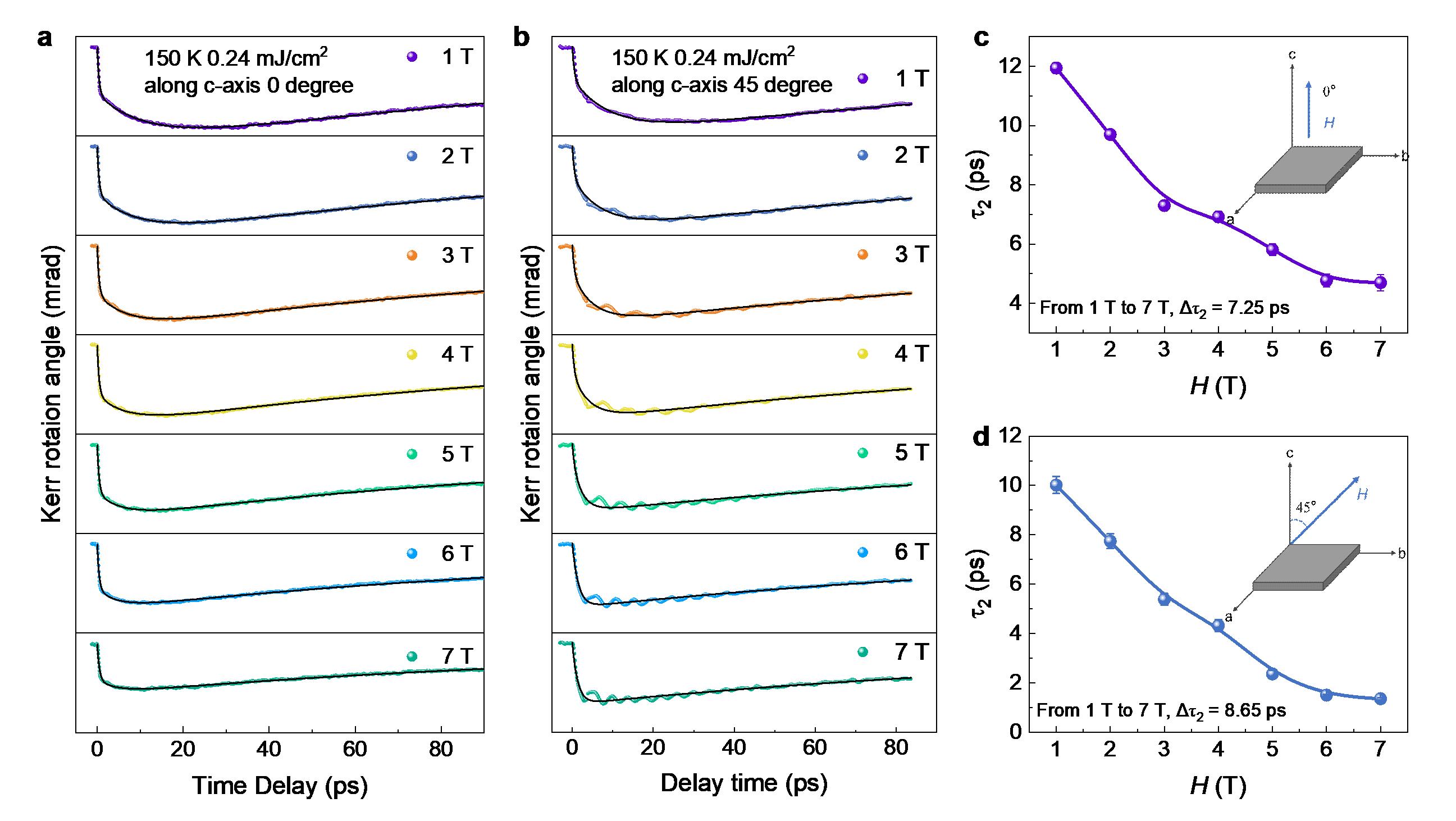


**Figure S6.** (a) and (b) Time-resolved Kerr rotation angle as a function of pump–probe delay time in thin-layer FGT flake under different magnetic field directions (along c-axis 0 and 45 degrees) at 150 K. (c) and (d) Magnetic field-dependence of *τ*_2_ under different magnetic field directions (see inset) extracted from the fit shown in the original data (a) and (b).


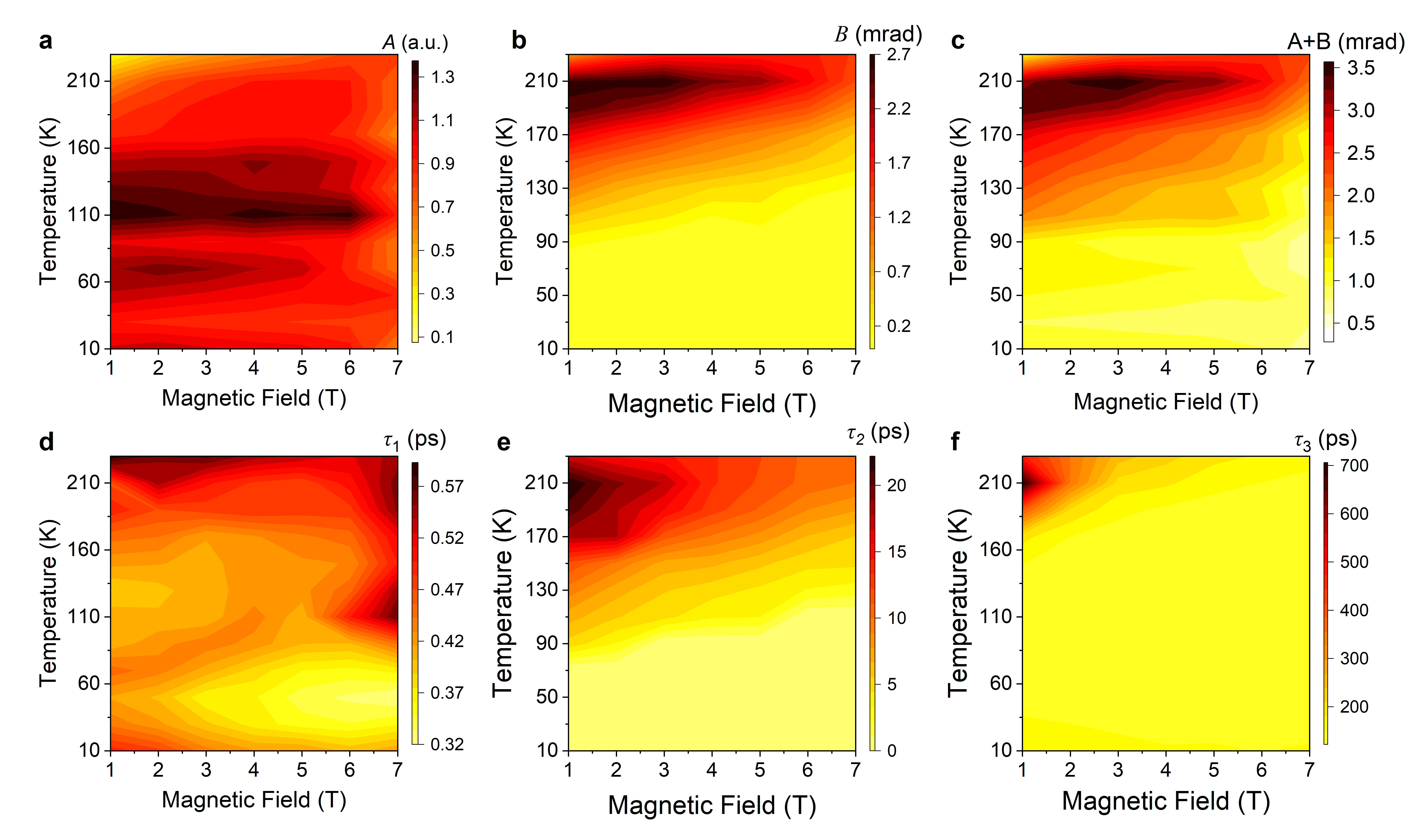


**Figure S7.** The temperature-magnetic field phase diagrams of *A*, *B*, *A*+*B*, *τ*_1_, *τ*_2_, and *τ*_3_ extracted from the fit shown in the original data.


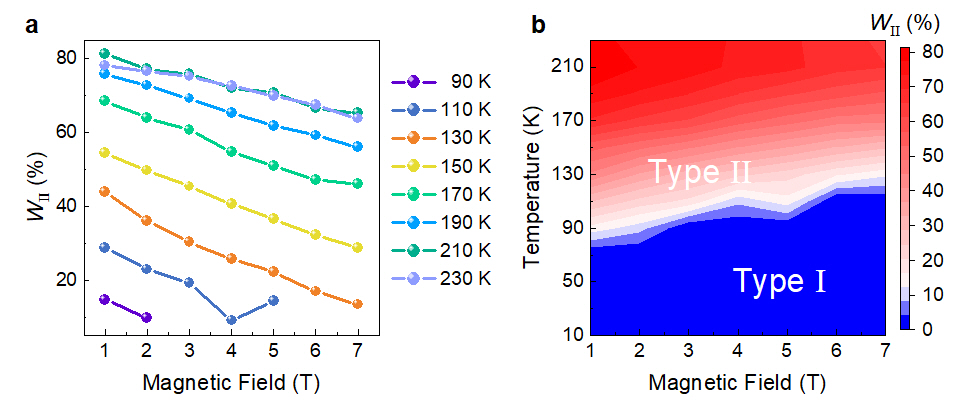


**Figure S8.** (a) Magnetic field dependence of *W*_Ⅱ_ calculated from the fitting values at selected temperature. (b) Temperature-magnetic field phase diagrams of *W*_Ⅱ_ calculated from the fitting values.
